# Evaluating IL-6 and IL-10 as rapid diagnostic tools for Gram-negative bacteria and as disease severity predictors in pediatric sepsis patients in the intensive care unit

**DOI:** 10.3389/fimmu.2022.1043968

**Published:** 2022-12-05

**Authors:** Yunyu Zhang, Biru Li, Botao Ning

**Affiliations:** Department of Pediatric Intensive Care Unit, Shanghai Children’s Medical Center, School of Medicine, Shanghai Jiao Tong University, Shanghai, China

**Keywords:** sepsis, pediatric intensive care unit, interleukin-6, interleukin-10, Gram-negative bacterial infection

## Abstract

**Background:**

To explore the diagnostic performance of interleukin (IL)-6 and IL-10 in discriminating Gram bacteria types and predicting disease severity in intensive care unit (ICU)-hospitalized pediatric sepsis patients.

**Method:**

We retrospectively collected Th1/Th2 cytokine profiles of 146 microbiologically documented sepsis patients. Patients were categorized into Gram-positive (G+) or Gram-negative (G-) sepsis groups, and cytokine levels were compared. Subgroup analysis was designed to eliminate the influence of other inflammatory responses on cytokine levels.

**Results:**

After propensity score matching, 78 patients were matched and categorized according to Gram bacteria types. Compared with G+ sepsis, IL-6 and IL-10 were significantly elevated in G- sepsis (p < 0.05). Spearman test proved the linear correlation between IL-6 and IL-10 (r = 0.654, p < 0.001), and their combination indicators (ratio and differences) were effective in identifying G- sepsis. In the subgroup analysis, such cytokine elevation was significant regardless of primary infection site. However, for patients with progressively deteriorating organ function [new or progressive multiple organ dysfunction syndrome (NPMODS)], differences in IL-6 and IL-10 levels were less significant between G+ and G- sepsis. In the receiver operating characteristic (ROC) curves of the G- sepsis group, the area under the curve (AUC) value for IL-6 and IL-10 was 0.679 (95% CI 0.561–0.798) and 0.637 (95% CI 0.512–0.762), respectively. The optimal cutoff value for diagnosing G- sepsis was 76.77 pg/ml and 18.90 pg/ml, respectively. While for the NPMODS group, the AUC for IL-6 and IL-10 was 0.834 (95% CI 0.766–0.902) and 0.781 (95% CI 0.701–0.860), respectively.

**Conclusion:**

IL-6 and IL-10 are comparably effective in discriminating G+/G- sepsis in pediatric intensive care unit (PICU) patients. The deteriorated organ function observed in ICU patients reveals that complex inflammatory responses might have contributed to the cytokine pattern observed in severe sepsis patients, therefore confounding the discriminating efficacy of Th1/Th2 cytokines in predicting Gram bacteria types.

## Introduction

Sepsis is defined as life-threatening organ dysfunction caused by dysregulated host immune responses to infection (1). As recommended in the 2020 Surviving Sepsis Campaign (SSC) international management guidelines, septic shock patients were recommended to receive empirical broad-spectrum antimicrobial therapy within 1 h of recognition and switch to narrower antimicrobial therapy once pathogen results were available (2). Although clinicians express concerns on challenges confronted with clinical implementation, evidence-based studies support its importance based on mortality reduction (3, 4).

During sepsis, pathogen-associated molecular pattern (PAMP) interactions with host immune cells induce cascades of inflammatory responses essential for pathogen clearance, while immune dysregulation might contribute to organ dysfunction (5). Diverse PAMPs expressed by bacterial strains result in different host immune responses. Gram-positive (G+) bacteria bind to Toll-like receptor 2 (TLR-2)-expressing host cells *via* lipoteichoic acid and secrete pneumolysin that lead to extensive endothelial damage (6). Gram-negative (G-) bacteria bind to TLR-4-expressing endothelial cells to promote interaction with leukocytes (7). Recently, cytokine analysis demonstrated that interleukin (IL)-6, IL-10, and Tumor Necrosis Factor-α (TNF-α) were significantly elevated in pediatric hematology patients with G- bacteremia (8). Moreover, the combination of multiple cytokines based on optimal cutoff values was similarly effective in discriminating G- bacterial infection (9). In other inflammatory syndromes such as hemophagocytic lymphohistiocytosis, cytokine release syndrome, and neutropenic septic shock, Th1/Th2 cytokine profiles also exhibited a certain discriminating power (10, 11).

In this study, we aimed to explore the diagnostic performance of Th1/Th2 cytokines in discriminating Gram bacteria types in intensive care unit (ICU)-hospitalized sepsis patients. With evidence of bacterial infection confirmed by specimen culture, patients were categorized into G+ or G- bacteria-induced sepsis groups. Receiver operating characteristic (ROC) curves were used to evaluate the diagnostic performance. Other critical conditions [severe sepsis, septic shock, multiple organ dysfunction syndrome (MODS)] that influence cytokine levels were considered and compared between G+ and G- sepsis groups. Finally, the prognostic predictive power of cytokines was evaluated.

## Materials and methods

### Study design, settings, and patients

This retrospective study was carried out in a single-center pediatric intensive care unit (PICU) from November 2017 to December 2020, approved by the ethical committee of Shanghai Children’s Medical Center (Approval number: SCMCIRB-K2018030), and written informed consent was obtained from patients’ guardians prior to enrollment. All enrolled patients met the criterion of “single bacterial infection” and were diagnosed with sepsis. They gave specimens for cultures and underwent cytokine measurements and pathogenic screening tests within 1 h of ICU admission. Patients with clinical conditions such as viral and fungal infection, primary immune deficiency, primary hemophagocytic lymphohistiocytosis or cytokine release syndrome were excluded from this study. Patients who underwent operations or trauma within 7 days were excluded. Patients with prior exposure to corticosteroids, granulocyte colony-stimulating factor, or biological agents were excluded.

Microbiologically documented patients without infectious clinical signs were randomly enrolled and considered as the control group. These patients were not healthy controls, instead they were non-febrile, non-infectious, critically ill patients admitted to the PICU. All case group patients (n = 146) were categorized into subgroups: 1) bloodstream infection and non-bloodstream infection; 2) acute respiratory distress syndrome (ARDS) and non-ARDS; 3) new or progressive multiple organ dysfunction syndrome (NPMODS) and non-NPMODS; 4) severe sepsis and non-severe sepsis. Subgroup analysis aimed to eliminate the impact of other inflammatory responses on cytokine levels.

### Definition and diagnostic criteria

With improved understanding of the sepsis pathobiology, the definition of Sepsis-3 and Sequential Organ Failure Assessment (SOFA) score were expected to be widely adopted in patients with suspected infection. However, there is a lack of diagnostic criterion and modified Pediatric Sequential Organ Failure Assessment (pSOFA) for pediatric population. In this study, clinical sepsis-related diagnosis was based on the 2015 pediatric septic shock expert consensus announced by the Chinese Pediatric Society, Chinese Medical Association. This clinical consensus was widely abided by pediatricians in Mainland China and was formulated based on the quantitative indicators of pediatric sepsis mentioned in the 2012 SSC international guidelines for management of severe sepsis and septic shock (12). Pediatric Risk of Mortality III (PRISM-III) was calculated using the worst physiological and laboratory indicators collected within 24 h of admission (13). The ARDS diagnosis follows the 2015 International Expert Consensus on Pediatric Acute Respiratory Distress Syndrome (14). NPMODS was defined as new-onset organ dysfunction or progressive deterioration of injured organ within 7 days of sepsis diagnosis (15). Organ function was assessed dynamically within admission day 1 to day 7.

Microbiological diagnosis is based on specimen cultures. For patients with more than one positive result, we only chose the predominating bacterial strain [(higher colony forming unit CFU)] and preferred results obtained from sterile specimens (blood, cerebrospinal fluid). The microbiological diagnosis confirmed by other pathogen tests was considered as mixed infection. Based on these rules, 14 patients (9.6%) had a positive blood culture associated with other body fluid cultures. All of these patients had the same bacteria isolated in both cultures.

### Blood sampling and cytokine measurement

Blood samples (2 ml each) were obtained from patients within 1 h of PICU admission and stored in tubes containing potassium EDTA at room temperature. Samples were immediately sent to the clinical laboratory to perform cytokine measurements. The cytometric bead array (CBA) was used as the cytokine detection methodology, with BD™ CBA Human Th1/Th2/Th17 cytokine kit II (from BD Biosciences) for cytokine measurement. Before CBA assays begin, negative controls and standards diluted in serial concentrations were prepared for the purpose of standard curve for calibration and validation of linearity. Bead populations were mixed, resuspended (mixed with serum enhancement buffer after the supernatant was removed), and added into assay tubes accordingly. For samples with a known high cytokine concentration, samples were diluted to obtain several desired dilutions with the assay diluent. Bead populations ready in a filter plate were mixed with samples to form the bead array that resolved in a red channel of the flow cytometer.

For the principles of CBA, beads conjugated with specific antibodies (with discrete fluorescence intensities) were captured and incubated with detection reagent and test samples to form sandwich complexes (beads + detection reagent + analyte). Such complexes provide proportional fluorescent signal relative to the concentration of that cytokine. Compared with conventional ELISA, flow cytometry could perform multi-analyte assays to detect series of cytokines by acquiring one single sample. Finally, Th1/Th2 cytokine levels were then quantitatively determined by a flow cytometer (FACSCalibur, BD Biosciences), and graphical results were generated by FCAP Array™ software. For the clinical reference range, upper normal limits for IL-2, IL-4, IL-6, IL-10, TNF-α, and Interferon-γ (IFN-γ) are 8.84, 3.88, 15.80, 4.98, 5.97, and 16.20 pg/ml, respectively.

### Statistical analysis

Statistical significance between categorical variables was compared using the Fisher’s exact test or chi-square (χ^2^) test, while the Mann–Whitney U test was used for skewed distribution continuous variables. Propensity score matching (PSM) was applied to balance baseline differences including comorbidities, PRISM-III, and critical condition (septic shock, severe sepsis) between the two groups. Match tolerance was 0.4. ROC curves and Youden index were used to determine diagnostic performances of clinical indicators. The correlation between IL-6 and IL-10 was compared using Spearman rank correlation analysis, while a binary logistic regression model was used to calculate the incidence of NPMODS. A two-sided p-value of <0.05 was considered statistically significant.

## Results

### Patient characteristics

A total of 226 patients were enrolled, of whom 146 sepsis patients with microbiologically documented infection comprised the case group and 80 non-infectious patients belonged to the control group. According to specimen culture results, 89 patients (61%) were categorized as G- sepsis, while 57 patients (39%) had G+ sepsis. PSM was used to balance the baseline characteristics. Finally, 78 matched patients were eligible for statistical analysis. As shown in **Table 1**, baseline characteristics were comparable between the two groups (p > 0.05). In our study, median age was 1 year 11 months, and patients from the preschool age group (38.5%) accounted the most **(** **Table 1** **)**. Most patients (51.3%) were admitted from the emergency department, followed by hospital transfer (24.4%) or general wards (24.4%). Comorbid condition was particularly prevalent (93.6%) with no significant differences between G+ and G- sepsis (p = 0.123). Nearly half of the patients (56.5%) had accompanying hematologic malignancies or under post-liver transplant/post-hematopoietic stem cell transplantation (HSCT) state. Other underlying comorbidities include cardiovascular (19.2%), hepatobiliary (9%), neuromuscular (3.8%), and respiratory diseases (2.6%) and inherited metabolic disorders (2.6%).

**Table 1:** Baseline information of Gram-positive and Gram-negative sepsis patients (after propensity score matching).

Characteristics	G+ sepsis(n=39)	G- sepsis(n=39)	All patients(n=78)	Z value/χ^2^	p value
**Age, [Median (range)]**	1year 5months (1month-13 years)	2years 8months (2months-16 years)	1year 11months (1 month-16 years)	-1.705	0.088
**Age group (%)**					
Infancy (29 days-12months)	17 (43.6)	10 (25.6)	27 (34.6)	3.446	0.352
Pre-school (1-5 years)	14 (35.9)	16 (41.0)	30 (38.5)		
School-aged (6-12 years)	6 (15.4)	8 (20.5)	14 (17.9)		
Adolescent (13-18 years)	2 (5.1)	5 (12.8)	7 (9.0)		
**Male (%)**	18 (46.2)	26 (66.7)	44 (56.4)	3.337	0.068
**Types of admission (%)**					
Emergency	25 (64.1)	15 (38.5)	40 (51.3)	5.132	0.077
General wards	7 (17.9)	12 (30.8)	19 (24.4)		
Other hospitals	7 (17.9)	12 (30.8)	19 (24.4)		
**Disease severity**					
PRISM-III scoring 1-10 (%)	28 (71.8)	29 (74.4)	57 (73.1)	0.129	0.938
PRISM-III scoring 11-20 (%)	6 (15.4)	6 (15.4)	12 (15.4)		
PRISM-III scoring>20 (%)	5 (12.8)	4 (10.3)	9 (11.5)		
**Types of comorbid condition (%)**					
With comorbid condition	37 (94.9)	36 (92.3)	73 (93.6)	0.215	0.643
Hematologic malignancy	10 (25.6)	15 (38.5)	25 (32.1)	10.405	0.123
Liver transplant or HSCT	7 (17.9)	12 (30.8)	19 (24.4)		
Cardiovascular	12 (30.8)	3 (7.7)	15 (19.2)		
Hepatobiliary	3 (7.7)	4 (10.3)	7 (9.0)		
Neuromuscular	2 (5.1)	1 (2.6)	3 (3.8)		
Respiratory	2 (5.1)	0 (0)	2 (2.6)		
Inherited metabolic disorder	1 (2.6)	1 (2.6)	2 (2.6)		
**Sources of culture specimen (%)**					
Respiratory tract	19 (48.7)	20 (51.3)	39 (50.0)	2.639	0.543
Bloodstream	17 (43.6)	16 (41.0)	33 (42.3)		
Gastrointestinal	1 (2.6)	3 (7.7)	4 (5.1)		
Thoracic/abdominal cavity	2 (5.1)	0 (0)	2 (2.6)		
**Complication and treatment (%)**					
Septic shock	17 (43.6)	22 (56.4)	39 (50.0)	1.282	0.258
Severe sepsis	26 (66.7)	30 (76.9)	56 (71.8)	1.013	0.314
Mechanical ventilation	19 (48.7)	23 (59.0)	42 (53.8)	0.825	0.364
Vasoactive drug infusion	16 (41.0)	23 (59.0)	39 (50.0)	2.513	0.113

HSCT, hematopoietic stem cell transplantation; PRISM-III, Pediatric Risk of Mortality III; G+, Gram-positive bacteria; G-, Gram-negative bacteria.

The most common primary site of infection was the respiratory tract (50%), followed by bloodstream (42.3%), gastrointestinal (5%), and thoracic/abdominal infections (2.6%). Majority of the patients presented with a mild PRISM-III score (73.1%) at sepsis onset. Only 26.9% of the patients gained a severe or extremely severe PRISM-III score, which was milder on average than the overall PRISM-III score distribution observed before PSM. For common critical condition and advanced supportive care, no statistical differences were observed between G+ and G- sepsis groups (**Table 1**). In this study, 50% of patients experienced septic shock, while 71.8% of the patients met the criteria of severe sepsis. Such high incidence might be attributed to the high prevalence of comorbid conditions among the enrolled patients.

### Th1/Th2 cytokine levels between sepsis and non-infectious patients

**Figure 1:**
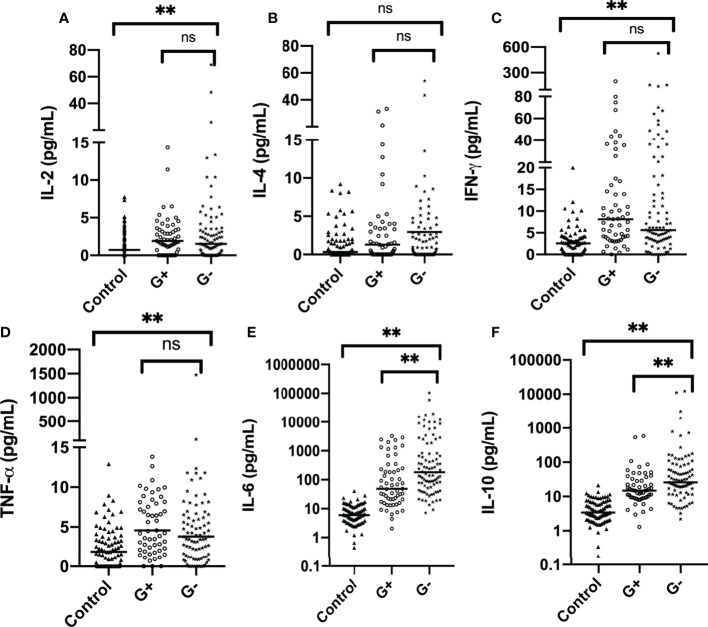
Distribution of Th1/Th2 cytokines in the control group and case group (before propensity score matching): **(A)** IL-2, **(B)** IL-4, **(C)** IFN-γ, **(D)** TNF-α, **(E)** IL-6, and **(F)** IL-10. The horizontal line represents the median level, while text brackets above represent p value. IL-6 and IL-10 are plotted with logarithmic scale. G-, Gram-negative bacteria; G+, Gram-positive bacteria; ns, p value with no significance, **p < 0.001. IL-2, interleukin-2; IL-4, interleukin-4; IFN-γ, interferon-γ; TNF-α, tumor necrosis-α; IL-6, interleukin-6; IL-10, interleukin-10.

Among all Th1/Th2 cytokines, IL-2, IL-6, IL-10, TNF-α, and IFN-γ were significantly elevated in infectious patients (p < 0.001). This supports the assumption that significant elevation of Th1/Th2 cytokines should be observed in febrile infectious patients (**Figures 1A** **–F**) . IL-2, IL-4, TNF-α, and IFN-γ were all slightly elevated, while IL-6 and IL-10 exhibited an exponential elevation proven by the logarithmic transformation scale (**Figures 1E, F** **)**. Medians of IL-6 levels in G+ and G- sepsis groups were 54.24 and 271.37 pg/ml, respectively. While medians of IL-10 in the two groups were 15.55 and 23.95 pg/ml, respectively (**Supplementary Table S1**). This indicated that more than half of the G- sepsis patients had their IL-6 exceeding 270 pg/ml, while the remaining 30.8% patients had IL-6 levels exceeding 1,000 pg/ml. In the G+ sepsis group, IL-6 levels only exceeded 270 pg/ml in 25.6% of the patients, while 15.4% of patients exceeded 1,000 pg/ml.

### Subgroup analysis of bloodstream infection and disease severity

In earlier studies, bacteremia is often the fundamental inclusion criterion, as it is the most common cause of severe sepsis in children with hematologic malignancies. However, few studies have investigated cytokine patterns in non-bloodstream infectious patients. In most ICUs, non-bloodstream infection dominates and possesses equal risk in developing septic shock and MODS. Therefore, it is necessary to set up subgroups for analysis. ARDS is a common complication in progressively deteriorating critically ill patients. Although the precipitating cause and pathophysiology of ARDS are complex, central to its pathogenesis is acute lung inflammatory injury. Involvement of robust inflammatory processes urges the setup of ARDS subgroups.

Distribution patterns of IL-6 and IL-10 in G+/G- sepsis patients were significantly different in both bloodstream and non-bloodstream infections (p < 0.05) (**Supplementary Table S2**). Discrepancies in the extent of IL-6 and IL-10 elevations were observed between subgroups of the same bacterial Gram types. For instance, in G- bacterial infection, IL-6 levels in bloodstream infection could reach 1,000–1,200 pg/ml while IL-6 levels in the corresponding non-bloodstream infection was barely 200 pg/ml. However, in the same subgroup, differences in cytokine levels between G+ and G- sepsis still exist. This supports the hypothesis that the discriminating power of IL-6 and IL-10 for G-/G+ sepsis is independent of the site of infection (bloodstream or non-bloodstream infection).

Regarding combined indicators, “IL-6 IL-10 differences” were statistically different between bacterial Gram types in the two subgroup analyses (**Supplementary Tables S2**, **S3**). G- sepsis patients showed relatively higher “IL-6 IL-10 differences.” This suggests that the extent of IL-6 and IL-10 elevation in G- sepsis patients was comparable regardless of site of inflammatory responses. However, “IL-6 IL-10 ratio” was relatively lower in G- sepsis patients with bloodstream infection and ARDS. While in the non-bloodstream and non-ARDS group, “IL-6 IL-10 ratio” was higher in G- sepsis patients but without statistical significance. One possible explanation for this adverse result might be the existence of other inflammatory responses that leads to a distinct IL-6–IL-10 variation pattern within bloodstream infection and ARDS patients.

Next, we evaluated the influence of critical conditions on Th1/Th2 cytokine levels by categorizing infectious patients into four subgroups: severe sepsis, non-severe sepsis, NPMODS, and non-NPMODS. In severe sepsis patients (n = 73) (**Supplementary Table S4**), no statistical significance was observed except for IL-10 (p = 0.034). This was consistent with a previous perspective stating that IL-10 was the most useful predictor for G- bacterial infection in critically-ill sepsis patients (16). Although the extent of the elevation was lesser than that observed in severe sepsis, cytokine patterns in G- bacterial infection with non-severe sepsis (n = 37) were comparable to those observed in case group analysis (p < 0.05).

In both NPMODS and non-NPMODS subgroups, median levels of IL-6 and IL-10 in patients with G- sepsis were higher than those with G+ sepsis (**Supplementary Table S5**). In the NPMODS subgroup, however, only IL-10 exhibited statistical differences between G+ and G- bacterial infection (p < 0.005), while IL-6 was significantly elevated regardless of bacterial Gram type (**Supplementary Table S5**).

### Combined indicators of IL-6 and IL-10 in Gram positive and Gram negative sepsis

**Figure 2:**
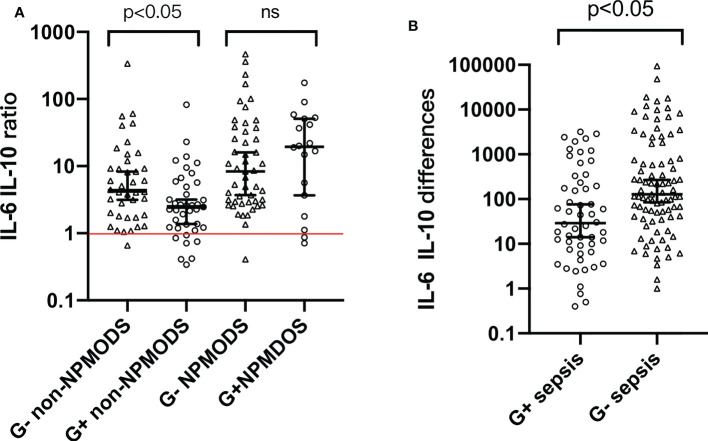
**(A)** Distribution pattern of “IL-6 IL-10 ratio” among G+ and G- sepsis patients. **(B)** Distribution pattern of “IL-6 IL-10 differences” among G+ and G- sepsis patients. Each symbol represents the combination indicator values of each individual in that group. Median and 95% confidence interval (95% CI) were shown using error bars. The red horizontal line in panel **(A)** represents “IL-6 IL-10 ratio = 1”. Brackets above bars represented the p value. ns, p value with no significance; G-, Gram-negative bacteria; G+, Gram-positive bacteria; NPMODS, new/progressive multiple organ dysfunction syndrome.

Based on the variation pattern of IL-6 and IL-10, we used combined indicators “IL-6 IL-10 ratio” and “IL-6 IL-10 differences” to further describe cytokine patterns among G+/G- sepsis patients (**Supplementary Table S1**). As shown in **Figure 2**, both indicators varied in their limited range within G+/G- sepsis groups with statistical significance (p < 0.05) (**Supplementary Table S1**). “IL-6 IL-10 ratio” represents the extent of IL-6 elevation and its fixated correlation with IL-10. “IL-6 IL-10 differences” further emphasized the prominent elevation. Almost all G- sepsis patients experience a marked elevation of IL-6 compared to IL-10 at admission (**Figure 2A**), which was less commonly observed in the G+ sepsis group.

For non-NPMODS patients, “IL-6 IL-10 ratio” after G- bacterial infection was statistically higher (p < 0.05) with a non-overlapping confidence interval (4.295, 95% CI 3.16–8.27) compared to G+ bacterial infection (2.470, 95% CI 1.37–3.18) (**Figure 2A**). For G- NPMODS sepsis patients, “IL-6 IL-10 ratio” varied in a narrow range similar to that observed in G- non-NPMODS sepsis. In contrast, G+ NPMODS sepsis patients exhibited dispersed levels that implied an inconsistent relationship between IL-6 and IL-10 in such group of patients. Most G+ NPMODS sepsis patients experienced significantly higher levels of pro-inflammatory IL-6 without relevant IL-10 elevation.

Next, we look at the usefulness of “IL-6 IL-10 differences” (**Figure 2B**). For G- sepsis patients, the observed narrower confidence intervals (compared to G+ sepsis patients) in “IL-6 and IL-10 differences” proved the efficacy of using such indicator to describe the IL-6–IL-10 correlation. Similarly, a statistical difference was observed between Gram bacteria types (p < 0.05).

**Figure 3:**
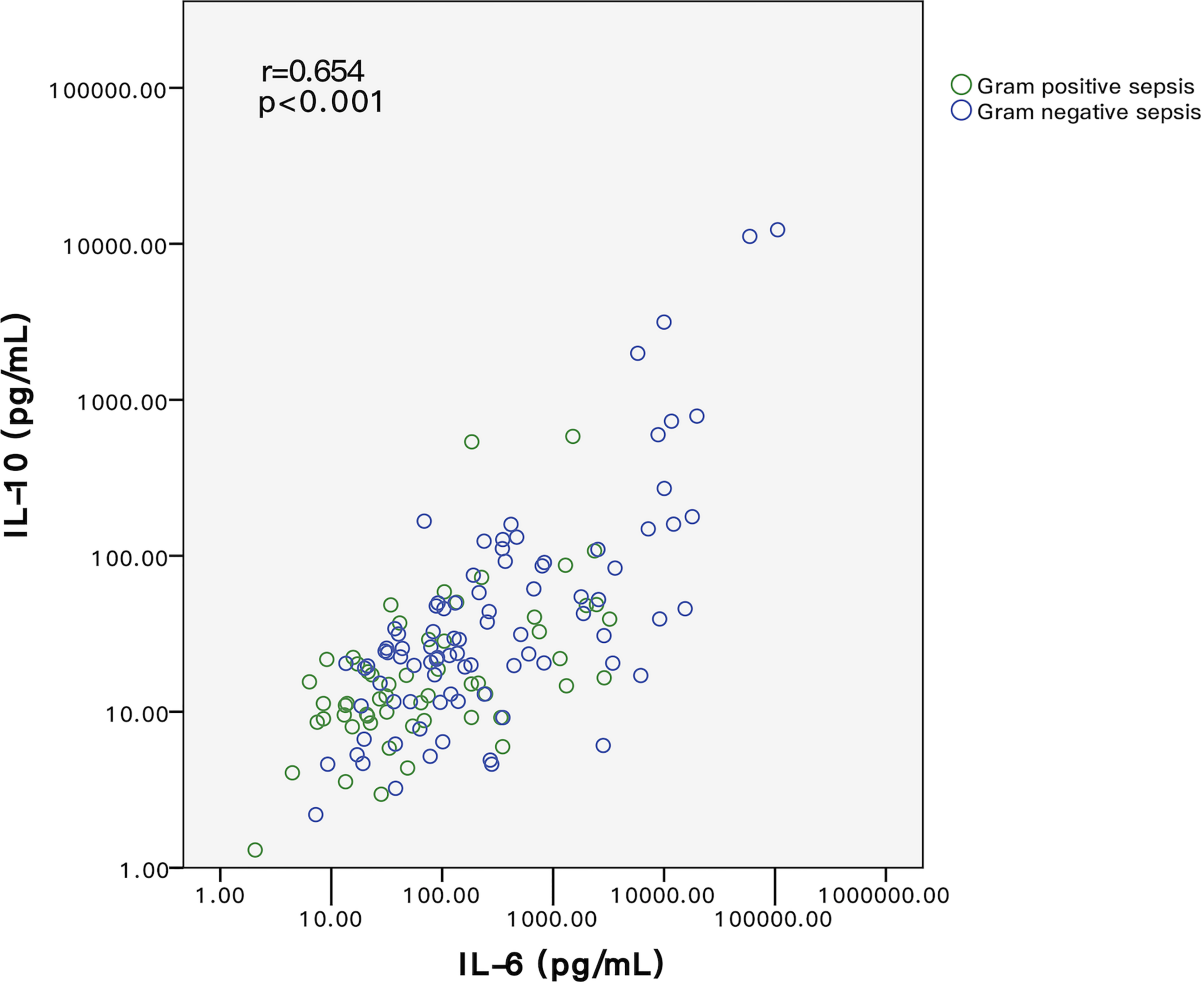
Relationship of IL-6 and IL-10 between G+ and G- sepsis patients. Each circle represents the measured IL-6 and IL-10 values of each individual. Spearman correlation rank test indicated that IL-6 and IL-10 were positively correlated. Among all patients, IL-6 and IL-10 levels in G- sepsis patients (blue circle) were significantly higher than those in G+ sepsis (green circle). G-, Gram-negative bacteria; G+, Gram-positive bacteria.

Binary scatter plots in **Figure 3** further proved a monotonic linear relationship between IL-6 and IL-10 among G+ and G- sepsis patients. In Spearman correlation rank analysis, IL-6 was positively correlated with IL-10 (r = 0.654, p < 0.001) (**Figure 3**), in which G- sepsis patients showed a stronger linear correlation between the two cytokines.

### Diagnostic performances of IL-6 and IL-10 in identifying Gram negative sepsis

**Figure 4:**
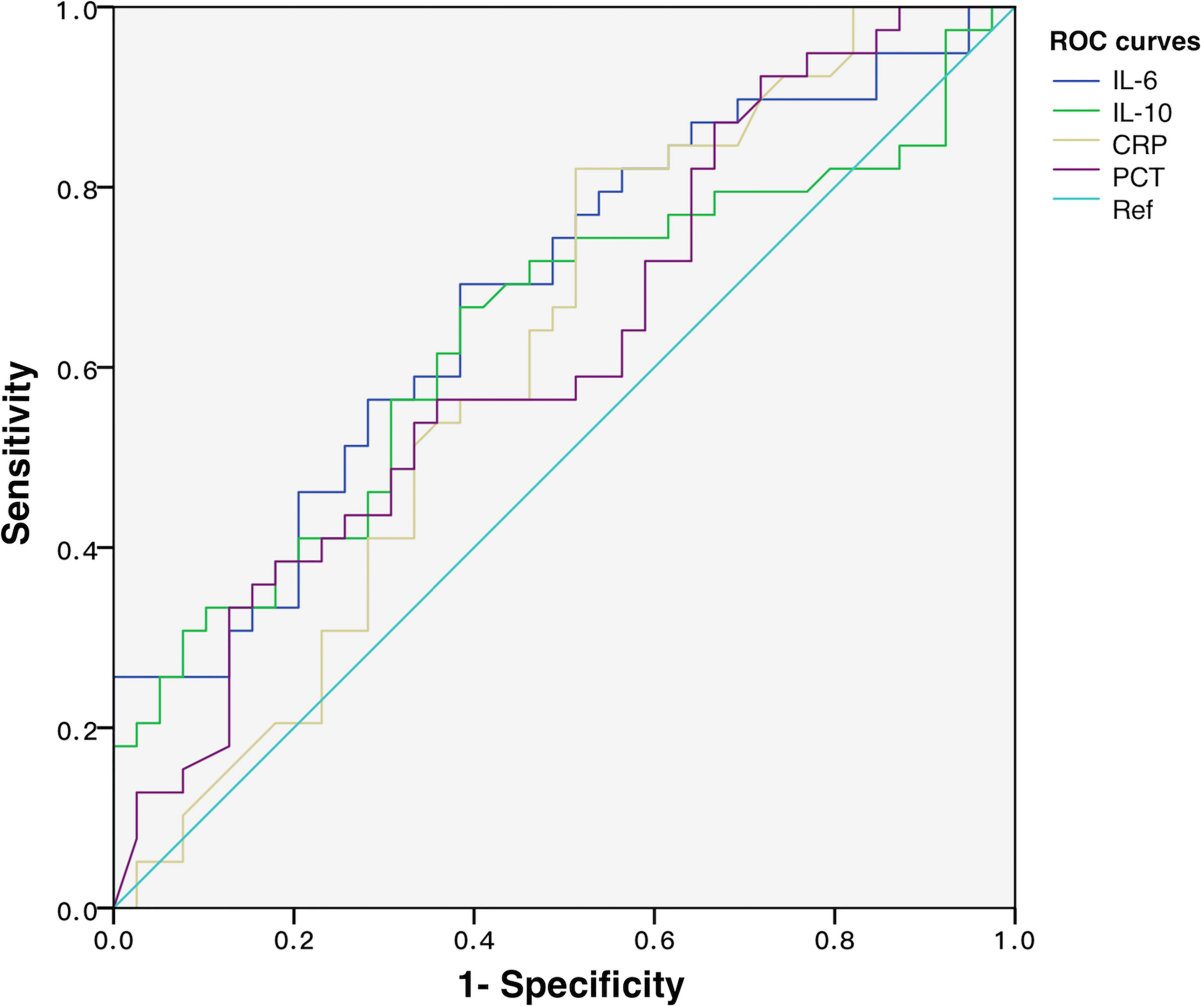
ROC curves of IL-6, IL-10, CRP, and PCT for discriminating Gram-negative bacteria-induced sepsis.

To discover the diagnostic performances of IL-6 and IL-10 in identifying G- bacterial infection, ROC curves were built based on IL-6, IL-10, C-reactive protein (CRP), and procalcitonin (PCT) of all of the matched patients (n = 78) (**Figure 4**). Area under the curve (AUC) values of IL-6, IL-10, C-reactive protein (CRP), and procalcitonin (PCT) were 0.679 (95% CI 0.561–0.798), 0.637 (95% CI 0.512–0.762), 0.616 (95% CI 0.489–0.742), and 0.623 (95% CI 0.499–0.747), respectively (**Table 2**). AUC values of IL-6 and IL-10 were superior than traditional indicators. Using Youden index, the optimal cutoff values of IL-6 and IL-10 were 76.77 and 18.90 pg/ml, in which specificity was 61.5% while sensitivity was 69.2% and 66.7%, respectively.

**Table 2:** Diagnostic performance of IL-6, IL-10, CRP, and PCT in discriminating Gram-negative sepsis and predicting NPMODS.

Indicator	Discriminating Gram-negative sepsis				Predicting new/progressive MODS			
	AUC	Cutoff	Sensitivity	Specificity	AUC	Cutoff	Sensitivity	Specificity
IL-6 (pg/ml)	0.679	76.77	69.2	61.5	0.834	187.93	73.0	81.3
		187.27	56.4	71.8		213.37	71.4	82.7
		249.05	51.3	74.4		308.96	65.1	89.3
IL-10 (pg/ml)	0.637	18.90	66.7	61.5	0.781	21.82	76.2	72.0
		20.40	61.5	64.1		25.78	71.4	81.3
		22.40	56.4	69.2		29.33	66.7	82.7
CRP (mg/L)	0.616	32.50	82.1	48.7	0.766	50.00	81.0	58.7
		36.50	76.9	48.7		61.50	74.6	66.7
		83.70	51.3	66.7		77.50	68.3	70.7
PCT (ng/ml)	0.623	4.29	56.4	64.1	0.725	5.32	55.6	78.7
		5.10	53.8	66.7		7.78	47.6	89.3
		5.32	51.3	66.7		8.13	46.0	90.7

AUC, area under the curve; MODS, multiple organ dysfunction syndrome; IL-6, interleukin-6; IL-10, interleukin-10; CRP, C-reactive protein; PCT, procalcitonin. The underlined cutoff value represents the optimal cutoff value for each indicator.

### Diagnostic performances of IL-6 and IL-10 on predicting new/progressive MODS

**Figure 5:**
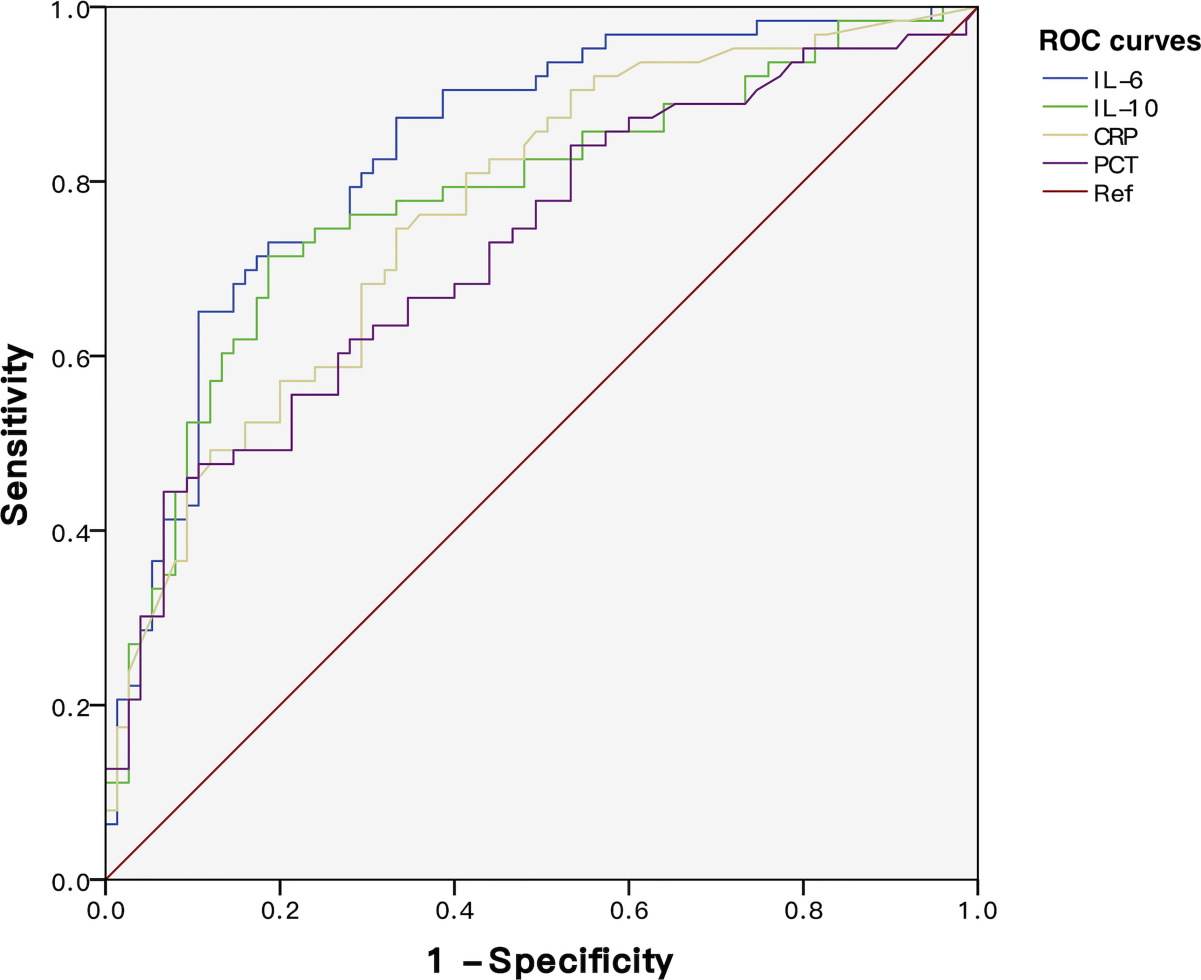
ROC curves of IL-6, IL-10, CRP, and PCT for predicting NPMODS. NPMODS, new/progressive multiple organ dysfunction syndrome.

To explore the predictive power of cytokines on sepsis prognosis, patients in the NPMODS subgroup (n = 67) were separately analyzed. ROC curves were shown in **Figure 5**, in which the AUC values for IL-6, IL-10, CRP, and PCT were 0.834 (95% CI 0.766–0.902), 0.781 (95% CI 0.701–0.860), 0.766 (95% CI 0.688–0.845), and 0.725 (95% CI 0.639–0.810), respectively. IL-6 and IL-10 showed remarkably higher AUC and non-overlapping optimal cutoff values (**Table 2** **)**. Although cytokines were measured at early onset of sepsis, IL-6 and IL-10 were sufficient enough for the prediction of NPMODS, in which their optimal cutoff values outperform that observed in diagnosing G- sepsis.

### Association of IL-6 and IL-10 with new/progressive MODS incidence

Binary logistic regression model was used to investigate the association of IL-6 and IL-10 with NPMODS incidence. Both cytokines were transformed into categorical variables presented as percentiles, in which the <P^25^ was used as a reference for comparison. Factors such as age, gender, bacteria types, and underlying diseases were included for adjustment. In this model, elevation of IL-6 and IL-10 showed a linear relationship with increased risk of NPMODS incidence (**Table 3**). With elevated levels of IL-6 and IL-10, the risk of NPMODS increased accordingly [higher odds ratio (OR)].

**Table 3:** Binary logistic regression model of IL-6 and IL-10 in predicting the incidence of NPMODS.

	B	SE	Wald	p value	Odds ratio	95% confidence interval
IL-6 (pg/ml)						
<104 (<25th)					1.00	
104-439 (25th–50th)	1.527	0.492	9.647	0.002	4.61	1.76-12.07
440-2,800 (50th–75th)	2.546	0.616	17.087	<0.001	12.76	3.82-42.66
>2,800 (>75th)	3.004	0.744	16.3	<0.001	20.17	4.69-86.74
IL-10 (pg/ml)						
<20 (<25th)					1.00	
20-46 (25th–50th)	0.706	0.467	2.284	0.131	2.03	0.81-5.06
47-110 (50th–75th)	2.351	0.603	15.213	<0.001	10.49	3.22-32.19
>110 (>75th)	3.104	0.829	14.016	<0.001	22.29	4.39-113.23

Based on cytokine levels observed in our study population, the 25th percentiles of IL-6 and IL-10 were used as reference for odds ratio calculation.

## Discussion

Previous studies mainly focused on patients with hematologic malignancies and have demonstrated the usefulness of cytokines for rapid identification of bacteremia Gram types (17). However, preliminary results might not work equally effectively in ICU scenarios, since the incidence of bacteremia, septic shock, or neutropenia was entirely different. Therefore, our study included critically ill sepsis patients regardless of underlying diseases. In consequence, their baseline physical status was more complicated, while comorbidities (93.6%) were more common than previously reported.

Among all Th1/Th2 cytokines, IL-6 and IL-10 were particularly elevated in G- sepsis patients. ROC curves demonstrated their improved diagnostic performance compared to traditional indicators (CRP and PCT). However, AUC values were less prominent, and cutoff values were relatively lower compared to those in previous studies. One reason might be the diverse disease complexity in ICU patients. Adjustment after PSM resulted in downsizing of the population. In consequence, the diagnostic performance might be underestimated by scattered outliers or confounding factors. All in all, Th1/Th2 cytokine patterns remain consistent with previous results and cutoff values work effectively in ICU settings. IL-6–IL-10 ratio and differences further demonstrated their linear correlation during G- bacterial infection. Previous studies have also focused on the correlation between “IL-6 and IL-10 ratio” and disease severity, proving its unique clinical value in early diagnosis of severe pneumonia (18). More importantly, “IL-6 IL-10 ratio” could easily reflect the extent of pro-inflammatory and anti-inflammatory status during sepsis, which is crucial in sepsis progression.

As a major pro-inflammatory cytokine, IL-6 recruits leukocytes and acute-phase proteins for inflammatory clearance processes. While the anti-inflammatory cytokine IL-10 regulates Th1/Th2 cell function and suppresses excessive pro-inflammatory factors (IL-6, IL-1, IL-8, TNF-α) mediated by the host response. In contrast, excessive IL-10 was associated with T-cell apoptosis and persistent infection. Inhibition of IL-6 expression by IL-10 might result in host immune paralysis. As previously reported, early elevation of IL-10 in G- bacteremia was associated with poor prognosis (19). In this situation, reduced inflammation fails to promote microbial clearance as the stage of infection is too early for B lymphocyte-mediated antibody-specific responses to take place (20). IL-6 was substantially secreted by macrophages and endothelial cells under inflammatory stimuli. As a multifunctional cytokine, IL-6 is involved not only in B-cell differentiation and T-cell activation but also in the crosstalk with pathological processes such as persistent complement activation and innate immune cell recruitment, cell apoptosis, and pyroptosis. Clinically, high levels of IL-6 were positively associated with disease severity and poor outcomes. To date, several studies have explored the diagnostic power of early IL-6 and IL-10 in severe infectious patients. Vanska et al. (21) found that IL-10 combined with PCT was effective in predicting the severe course of febrile neutropenia. Persson et al. (22) suggested that lower IL-6 levels in the early stage of neutropenic fever could effectively predict a mild infection.

Subgroup analysis aimed to eliminate the influence of infection site and critical condition on cytokines. IL-6 and IL-10 levels between Gram bacteria types in severe subgroups (bloodstream infectious patients with higher serum cytokines and sepsis patients with ARDS) might reflect the existence of other inflammatory responses that is independent of the initial bacterial infection. Indeed, IL-6 and IL-10 levels were influenced not only by bacterial Gram types but also by progressive organ dysfunction. Patients with severe sepsis and NPMODS were characterized by worsening organ function at early sepsis onset. Although IL-6 and IL-10 were significantly higher in patients with severe sepsis and NPMODS than those in the non-severe sepsis group, no significant IL-6 differences between G+ and G- sepsis were observed, in which cytokine levels during the critical stage might be independent of Gram bacteria type. While in patients without progressive organ failure, IL-6 and IL-10 levels were still significantly elevated after G- bacterial infection, proving the comparable G+/G- bacteria discriminating power in non-severe sepsis. With similar results observed in the non-NPMODS group, this further confirms that IL-6 elevation in critically ill patients was attributed to progressively worsening organ failure but independent of baseline organ dysfunction.

During progressive organ dysfunction, the presence of other pathophysiological processes might result in significant elevation of IL-6. IL-6 elevation observed here was attributed to organ failure but not bacterial Gram types. Similarly, previous studies found no independent association between causative organism, infection site, or bacteremia and sepsis mortality. When factors such as disease severity and organ dysfunction were taken into account, primary infection has a subtle impact on sepsis prognosis (23). These provided rationales for the weakened clinical importance of discriminating G+/G- sepsis among severe-onset patients. Although variations of IL-10 levels between subgroups were minor, differences in IL-10 levels were still statistically significant between bacterial Gram types, suggesting its unique power in identifying bacterial Gram types.

This study has several limitations. First, the sepsis population might be underestimated. In an earlier survey, bacterial infection only accounts for half (55%) of the ICU sepsis, while majority of the patients experience >1 pathogenic infection (24). Second, comorbid conditions might have exerted a certain effect on cytokine levels. More than half of the patients (56.5%) had accompanying hematologic malignancies or under post-transplant state. These patients who received immunosuppressive therapy might have developed secondary immunodeficiency that altered normal immune function, resulting in a distinct cytokine pattern. Third, clinical courses were inconsistent. Different admission routes, clinical presentation, and severity among ICU patients make it difficult to determine the actual clinical stage. Therefore, subgroup analyses were incapable of evaluating the dynamic changes of cytokines during the sepsis course. Fourth, sepsis patients lacking microbiological results were excluded. In a previous epidemiological survey, 82.6% of the patients have their specimen taken for culture at sepsis diagnosis (25), while the reported positive rate was not high (50.1%–65.4%) (26). Our study was incapable of including microbiologically undocumented sepsis patients.

To conclude, Th1/Th2 cytokines, especially IL-6 and IL-10, are comparably effective in discriminating G+/G- bacteria-induced sepsis in the PICU. Subgroup analysis revealed that for sepsis patients characterized by progressive organ dysfunction, levels of IL-6 and IL-10 were less helpful in identifying bacterial Gram types while more strongly correlated with NPMODS but independent of baseline organ dysfunction. Furthermore, IL-6 and IL-10 elevation at sepsis onset was associated with an increased risk of NPMODS, proving the efficacy of Th1/Th2 cytokines in predicting sepsis prognosis.

## Data availability statement

All datasets generated for this study are included in the article/**Supplementary Material**.

## Ethics statement

The studies involving human participants were reviewed and approved by ethical committee of Shanghai Children’s Medical Center. Written informed consent to participate in this study was provided by the participants’ legal guardian/next of kin.

## Author contributions

YZ and BN conceived and designed the study. YZ collected data, conducted data analysis, illustrated figures and drafted the manuscript. BN and BL helped to revise the draft of the manuscript. Final approval of the manuscript was confirmed by all authors.

## Funding

This study was supported by grants from the Shanghai Natural Science Foundation of China (grant No. 19ZR1432900) and Shanghai Translational Medicine Collaborative Innovation Center (grant No. TM202012).

## Acknowledgments

The authors would like to thank all the participants and guardians for their support on this clinical research work. We also like to thank the microbiology laboratory and molecular laboratory for providing fast accurate specimen culture and cytokine results.

## Conflict of interest

The authors declare that the research was conducted in the absence of any commercial or financial relationships that could be construed as a potential conflict of interest.

## Publisher’s note

All claims expressed in this article are solely those of the authors and do not necessarily represent those of their affiliated organizations, or those of the publisher, the editors and the reviewers. Any product that may be evaluated in this article, or claim that may be made by its manufacturer, is not guaranteed or endorsed by the publisher.
