# Supplementary material for: Evaluating IL-6 and IL-10 as rapid diagnostic tools for Gram-negative bacteria and as disease severity predictors in pediatric sepsis patients in the intensive care unit
Source: Front Immunol. 2022 Dec 5;13:1043968. doi: 10.3389/fimmu.2022.1043968 (PMC9760793; doi:10.3389/fimmu.2022.1043968)
Supplement: Supplementary file 1 [file Table_1.docx]

**Supplemental Digital Content**

Supplemental Table 1. Median (range) and statistical test results of Th1/Th2 cytokines between control group and case group (Gram positive group and Gram negative sepsis group).

| Groups | Control group | Case group | | Z value (p value)^*^ | Z value (p value)^#^ |
| --- | --- | --- | --- | --- | --- |
|  | Non-infectious  (n=80) | Gram positive sepsis  (n=39) | Gram negative sepsis  (n=39) |  |  |
| IL-2 (pg/mL) | 0.72 (0-7.76) | 2.04 (0-14.34) | 1.47 (0-48.46) | -2.939 (p<0.005) | ns |
| IL-4 (pg/mL) | 0.34 (0-9.21) | 1.03 (0-31.32) | 0.21 (0-8.59) | ns | ns |
| IL-6 (pg/mL) | 5.95 (0-41.0) | 54.24 (2.06-3234.48) | 271.37 (7.25-105695.33) | -10.006 (p<0.001) | -2.723 (p=0.006) |
| IL-10 (pg/mL) | 3.40 (0-21.31) | 15.55 (1.3-107.53) | 23.95 (2.19-12319.26) | -8.882 (p<0.001) | -2.084 (p=0.037) |
| TNF-α (pg/mL) | 1.88 (0-12.92) | 4.49 (0-22.05) | 4.28 (0-1477.33) | -5.313 (p<0.001) | ns |
| IFN-γ (pg/mL) | 2.56 (0-20.0) | 8.24 (0-198.54) | 6.18 (0-525.40) | -6.700 (p<0.001) | ns |
| IL6 IL-10 differences | / | 45.72 (0.76-3195.21) | 266.47 (4.68-93376.07) | / | -2.523 (0.012) |
| IL6 IL-10 ratio | / | 3.18 (0.41-174.88) | 8.11 (1.24-361.43) | / | -1.734 (0.042) |
| C-reactive protein (mg/L) | / | 46.00 (0-270) | 85.40 (5-262) | / | -1.760 (0.078) |
| Procalcitonin (ng/mL) | / | 2.36 (0-100) | 5.41 (0.06-100) | / | -1.864 (0.062) |

^*^=statistical significance between control group and case group, ^#^= statistical significance between Gram positive and Gram negative sepsis group, ns=no significance

Supplemental Table 2. Subgroup analysis: Comparison of cytokine levels between Gram positive and Gram negative sepsis patients with bloodstream infection or without bloodstream infection.

| Subgroup | Cases | IL-2 (pg/mL) | IL-4 (pg/mL) | IL-6 (pg/mL) | IL-10 (pg/mL) | TNF-α (pg/mL) | IFN-γ (pg/mL) | IL6 IL-10 differences | IL6 IL-10 ratio |
| --- | --- | --- | --- | --- | --- | --- | --- | --- | --- |
| 1. Bloodstream infection | |  |  |  |  |  |  |  |  |
| Gram positive sepsis | 24 | 1.79  (0-6.51) | 1.15 (0-31.32) | 292.19 (7.47-3234.48) | 15.16 (3.56-582.76) | 3.73 (0-21.93) | 4.37 (0-35.47) | 281.09 (1.10-3195.21) | 17.86 (0.87-174.88) |
| Gram negative sepsis | 34 | 1.59 (0-48.46) | 0.485 (0-10.27) | 1229.62 (9.29-105695.33) | 48.61 (3.23-12319.26) | 3.300 (0-1477.33) | 6.18 (0-156.88) | 1171.65 (4.68-93376.07) | 15.278 (1.72-465.48) |
| Z value |  | -2.90 | -0.710 | -4.400 | -2.87 | -1.875 | -2.03 | -3.874 | -3.374 |
| p value |  | 0.772 | 0.478 | <0.001 | <0.005 | 0.061 | 0.042 | <0.001 | <0.001 |
| 2.Non-bloodstream infection | |  |  |  |  |  |  |  |  |
| Gram positive sepsis | 33 | 2.04 (0-14.34) | 1.3 (0-33.28) | 28.2 (2.06-1292.49) | 12.67 (1.3-538.17) | 6.41 (0.72-22.05) | 10.66 (1.15-198.54) | 14.06 (0.4-1205.51) | 2.21 (0.34-14.86) |
| Gram negative sepsis | 55 | 1.53 (0-69.11) | 0.51 (0-54.14) | 91.75 (7.25-15501.3) | 22.49 (2.19-166.84) | 4.26 (0-124.69) | 5.38 (0-525.4) | 72.74 (1.01-15455.5) | 3.8031 (0.41-361.43) |
| Z value |  | -0.270 | -0.793 | -2.195 | -2.352 | -0.284 | -1.208 | -2.195 | -0.063 |
| p value |  | 0.787 | 0.428 | <0.05 | <0.05 | 0.776 | 0.227 | <0.05 | 0.95 |

Supplemental Table 3. Subgroup analysis: Comparison of cytokine levels between Gram positive and Gram negative sepsis patients with acute respiratory distress syndrome (ARDS) or without ARDS.

| Subgroup | Cases | IL-2 (pg/mL) | IL-4 (pg/mL) | IL-6 (pg/mL) | IL-10 (pg/mL) | TNF-α (pg/mL) | IFN-γ (pg/mL) | IL6 IL-10 differences | IL6 IL-10 ratio |
| --- | --- | --- | --- | --- | --- | --- | --- | --- | --- |
| 1.Acute respiratory distress syndrome (ARDS) | | | |  |  |  |  |  |  |
| Gram positive sepsis | 14 | 2.41 (0-5.38) | 1.01 (0-12.72) | 98.14  (7.47-3234.48) | 24.31  (8.57-538.17) | 3.83  (1.16-9.81) | 10.45  (0.65-79.73) | 74.58  (1.1-3195.21) | 4.29  (0.34-174.88) |
| Gram negative sepsis | 16 | 1.44  (0-69.11) | 0  (0-54.13) | 1070.56 (68.85-17964.55) | 82.24  (20.5-3151.51) | 4.52  (0-124.69) | 10.35  (1.89-140.02) | 979.71  (50.29-17786.38) | 4.04  (0.41-232.14) |
| Z value |  | -0.813 | -1.066 | -2.286 | -2.785 | -0.457 | -0.208 | -2.162 | -0.915 |
| p value |  | 0.423 | 0.334 | <0.05 | <0.005 | 0.667 | 0.854 | <0.05 | 0.377 |
| 2. Non-ARDS | | | |  |  |  |  |  |  |
| Gram positive sepsis | 43 | 1.80  (0-14.34) | 1.35  (0-33.28) | 33.35  (2.06-2362.73) | 12.67  (1.3-582.76) | 5.78  (0-22.05) | 7.30 (0-198.54) | 27.51  (0.4-2255.2) | 2.68  (0.41-89.95) |
| Gram negative sepsis | 73 | 1.54  (0-48.46) | 0.86  (0-43.54) | 139.31  (7.25-105695.33) | 22.95  (2.19-12319.26) | 3.52  (0-1477.33) | 5.06  (0-525.4) | 115.88  (1.01-93376.07) | 5.79  (0.66-465.48) |
| Z value |  | -0.02 | -0.738 | -3.364 | -3.055 | -1.738 | -1.086 | -2.89 | -2.106 |
| p value |  | 0.984 | 0.461 | <0.001 | <0.005 | 0.082 | 0.277 | <0.005 | 0.053 |

Supplemental Table 4. Subgroup analysis: Comparison of cytokine levels between Gram positive and Gram negative sepsis patients that developed severe sepsis or without severe sepsis.

| Subgroup | Cases | IL-2 (pg/mL) | IL-4 (pg/mL) | IL-6 (pg/mL) | IL-10 (pg/mL) | TNF-α (pg/mL) | IFN-γ (pg/mL) | IL6 IL-10 differences | IL6 IL-10 ratio |
| --- | --- | --- | --- | --- | --- | --- | --- | --- | --- |
| 1. Severe sepsis | |  |  |  |  |  |  |  |  |
| Gram positive sepsis | 21 | 2.77  (0-6.51) | 1.44  (0-17.9) | 338.26  (7.47-3234.48) | 21.92  (5.84-582.76) | 4.18  (0-21.93) | 6.69  (0-67.62) | 329.08  (1.1-3195.21) | 16.80 (0.71-174.88) |
| Gram negative sepsis | 52 | 1.47  (0-48.46) | 0.25  (0-8.92) | 556.87  (19.78-105695.33) | 44.23  (3.23-12319.26) | 3.49  (0-1477.33) | 5.67  (0-156.88) | 529.48  (0-93376.07) | 11.35  (0.41-465.48) |
| Z value |  | -0.802 | -2.195 | -1.523 | -2.114 | -0.981 | -0.195 | -0.122 | -1.475 |
| p value |  | 0.423 | 0.028 | 0.128 | 0.034 | 0.326 | 0.845 | 0.903 | 0.14 |
| 2. Non-severe sepsis |  |  |  |  |  |  |  |  |  |
| Gram positive sepsis | 36 | 1.79  (0-14.34) | 0.38  (0-33.28) | 27.75  (2.06-751.18) | 12.36  (1.3-72.73) | 5.18  (0-22.05) | 8.84  (0.43-198.54) | 16.65  (0.4-718.58) | 2.23 (0.34-58.77) |
| Gram negative sepsis | 37 | 1.56  (0-69.11) | 0.96  (0-54.13) | 77.92  (7.25-15501.3) | 20.42  (2.19-127.11) | 3.98  (0-124.69) | 5.42  (0-525.4) | 40.74  (1.01-15455.5) | 3.23 (0.66-338.46) |
| Z value |  | -0.416 | -0.519 | -2.461 | -2.223 | -0.916 | -0.684 | -1.688 | -1.732 |
| p value |  | 0.678 | 0.604 | 0.014 | 0.026 | 0.360 | 0.494 | 0.091 | 0.083 |

Supplemental Table 5. Subgroup analysis: Comparison of cytokine levels between Gram positive and Gram negative sepsis patients with NPMODS or without NPMODS.

| Subgroup | Cases | IL-2 (pg/mL) | IL-4 (pg/mL) | IL-6 (pg/mL) | | IL-10 (pg/mL) | | TNF-α (pg/mL) | | IFN-γ (pg/mL) | IL6 IL-10 differences | IL6 IL-10 ratio |
| --- | --- | --- | --- | --- | --- | --- | --- | --- | --- | --- | --- | --- |
| 1.New/progressive multiple organ dysfunction syndrome (NPMODS) | | | | |  | |  |  |  | |  |  |
| Gram positive sepsis | 18 | 1.98  (0-6.51) | 1.36  (0-10.45) | 344.55  (7.47-2885.51) | | 25.15  (5.97-107.53) | | 3.32  (0-21.93) | | 5.71  (0-67.62) | 336.97  (1.1-2869.01) | 19.44  (0.71-174.88) |
| Gram negative sepsis | 49 | 1.47  (0-69.11) | 0.34  (0-54.13) | 469.95  (19.78-105695.33) | | 52.45  (3.23-12319.26) | | 4.26  (0-1477.33) | | 5.71  (0-156.88) | 423.77  (8.28-93376.07) | 8.3612  (0.41-465.48) |
| Z value |  | -0.213 | -0.726 | -1.315 | | -2.794 | | -0.05 | | -0.035 | -1.231 | -0.82 |
| p value |  | 0.831 | 0.468 | 0.188 | | <0.005 | | 0.961 | | 0.972 | 0.412 | 0.218 |
| 2. Non-NPMODS |  |  |  |  | |  | |  | |  |  |  |
| Gram positive sepsis | 39 | 1.79  (0-14.34) | 1.03  (0-33.28) | 28.20  (2.06-3234.48) | | 12.64  (1.3-582.76) | | 6.60  (0-22.05) | | 8.64  (0.43-198.54) | 18.08  (0.4-3195.21) | 2.47  (0.34-82.37) |
| Gram negative sepsis | 40 | 1.62  (0-25.91) | 0.93  (0-43.54) | 70.43  (7.25-15501.3) | | 19.75  (2.19-109.89) | | 3.66  (0-26.83) | | 5.40  (0-525.4) | 56.70  (1.01-15455.5) | 4.30  (0.66-338.46) |
| Z value |  | -0.202 | -0.664 | -2.579 | | -1.378 | | -1.746 | | -0.937 | -1.902 | -2.491 |
| p value |  | 0.840 | 0.506 | <0.01 | | 0.168 | | 0.081 | | 0.349 | 0.057 | <0.05 |
